# Supplementary material for: “Force-From-Lipids” Dependence of the MscCG Mechanosensitive Channel Gating on Anionic Membranes
Source: Microorganisms. 2023 Jan 12;11(1):194. doi: 10.3390/microorganisms11010194 (PMC9861469; doi:10.3390/microorganisms11010194)
Supplement: Supplementary file 1 [file microorganisms-11-00194-s001.zip › Supplementary Materials-YN1.pdf]

Supplementary Materials

"Force-From-Lipids" Dependence of the MscCG Mechanosensitive Channel Gating on Anionic Membranes

Yoshitaka Nakayama[1, 2], Paul R. Rohde[1], Boris Martinac[1, 2, *]

[1] Molecular Cardiology and Biophysics Division, Victor Chang Cardiac Research Institute, Sydney 2010, Australia
[2] Faculty of Medicine, St Vincent's Clinical School, The University of New South Wales, Sydney 2010, Australia
***** Correspondence: b.martinac@victorchang.edu.au; Tel.: +61-2-9295-8743

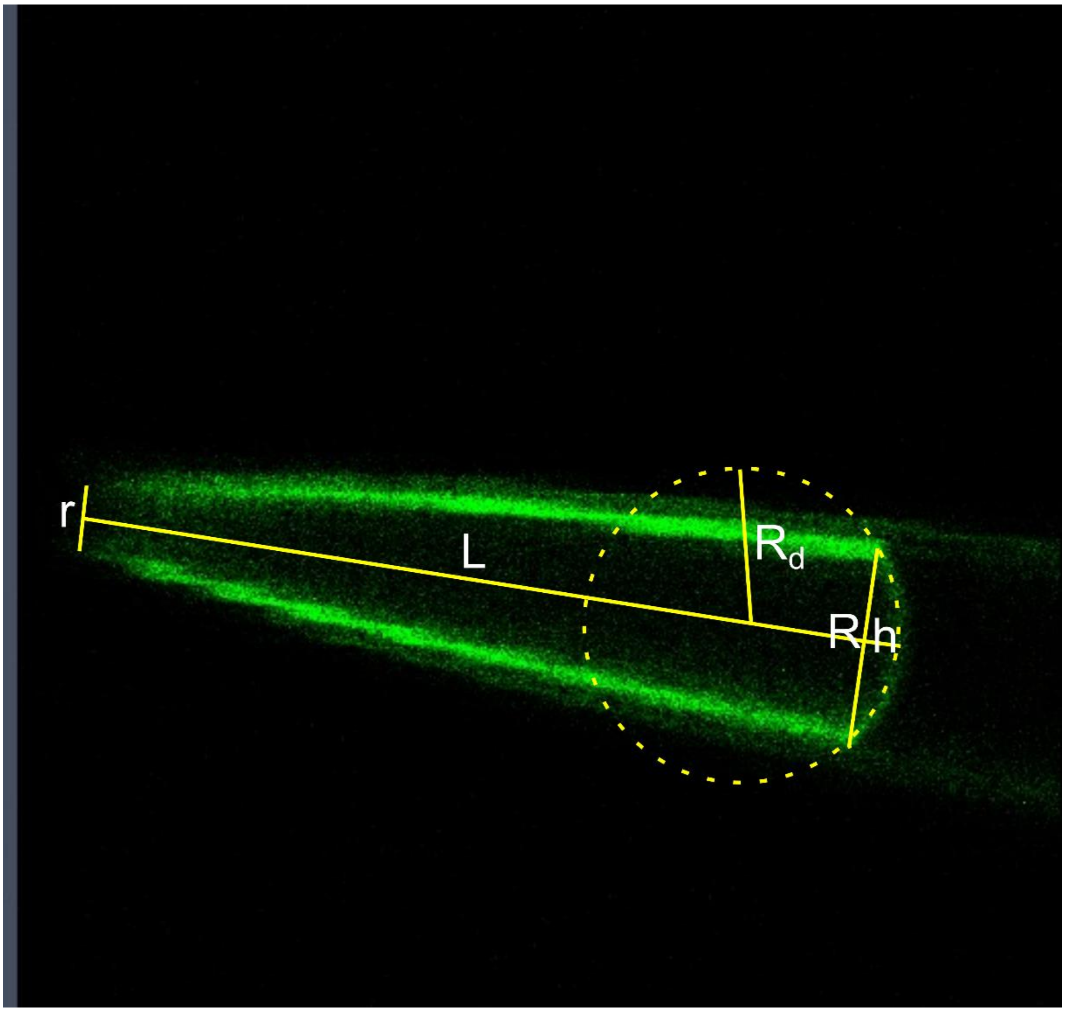

Figure S1 Parameters for the areal elasticity modulus K_A in fluorescent images of excised patch membranes. Membrane tension (T) was calculated based on Laplace's law with the radius of membrane curvature (R_d) under pressure. Membrane area (A) was calculated with the protrusion length (L), the pipette radius at the pipette tip (r), the pipette radius at the dome (R), and the height of the dome of the patch membrane (h).

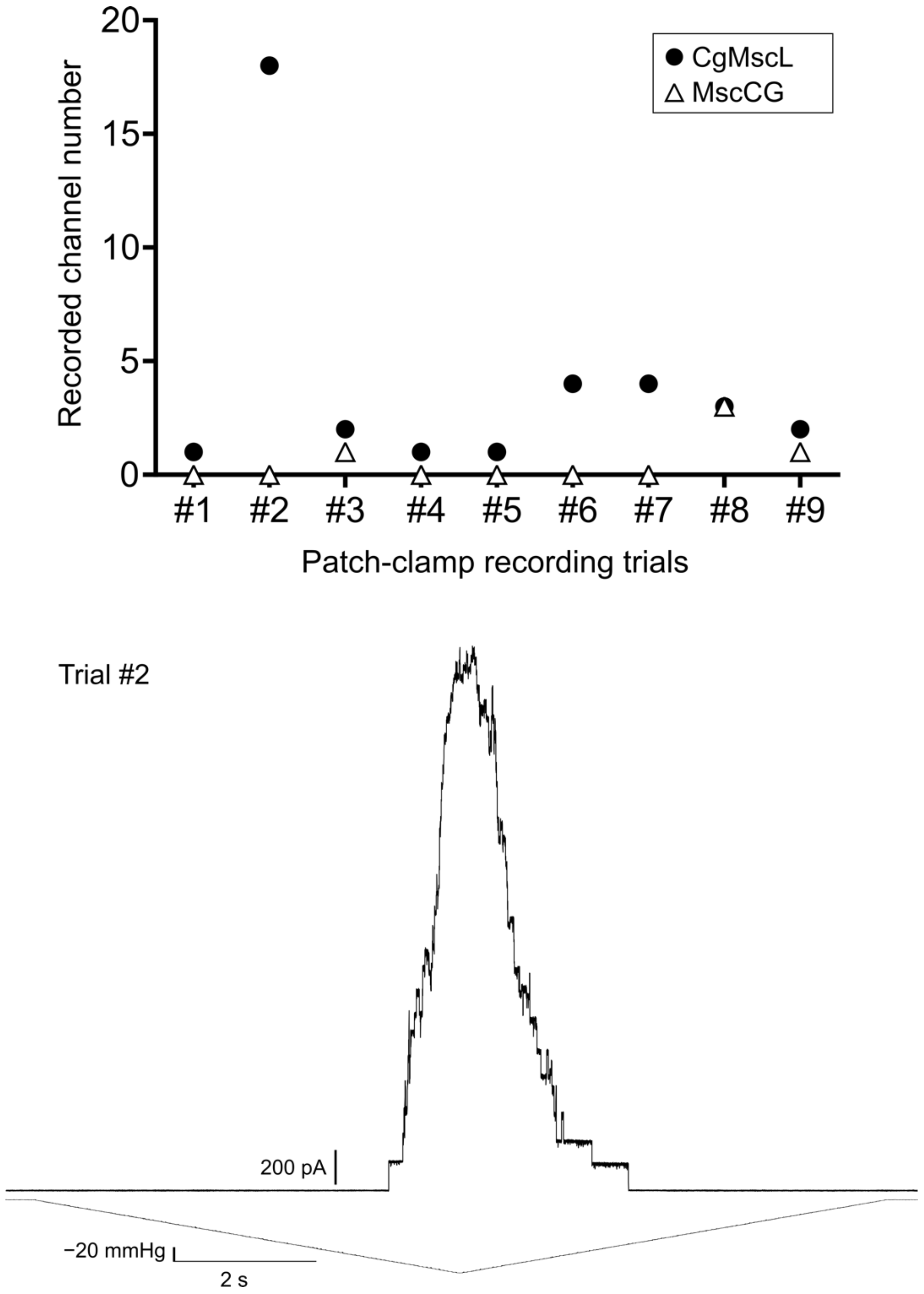

Figure S2 Channel distribution of MscCG and CgMscL at the native expression level in *C. glutamicum* membrane vesicle liposomes. Recorded channel number of MscCG and CgMscL in membrane vesicle liposomes without overexpression from nine different patches (Trials #1-9) (top). A recording of trial #2 shows outstanding number of CgMscL among trials (bottom).

Video S1 Liposomal membranes of the neutral DOPE/DOPC(70%:30%) applied by negative pressure steps increased by every –5 mmHg until –50 mmHg.

Video S2 Liposomal membranes of the negatively charged DOPE/DOPG(70%:30%) applied by negative pressure steps increased by every –5 mmHg until –50 mmHg.
